# Combining automatic plan integrity check (APIC) with standard plan document and checklist method to reduce errors in treatment planning

**DOI:** 10.1002/acm2.12981

**Published:** 2020-07-17

**Authors:** Ping Xia, Danielle LaHurd, Peng Qi, Anthony Mastroianni, Daesung Lee, Anthony Magnelli, Eric Murray, Matt Kolar, Bingqi Guo, Tim Meier, Samual T. Chao, John H. Suh, Naichang Yu

**Affiliations:** ^1^ Department of Radiation Oncology Taussig Cancer Institute Cleveland Clinic Cleveland OH 44195 USA

**Keywords:** automation, planning errors, radiotherapy, standardization, treatment plans

## Abstract

**Purpose/objectives:**

To report our experience of combining three approaches of an automatic plan integrity check (APIC), a standard plan documentation, and checklist methods to minimize errors in the treatment planning process.

**Materials/methods:**

We developed APIC program and standardized plan documentation via scripting in the treatment planning system, with an enforce function of APIC usage. We used a checklist method to check for communication errors in patient charts (referred to as chart errors). Any errors in the plans and charts (referred to as the planning errors) discovered during the initial chart check by the therapists were reported to our institutional Workflow Enhancement (WE) system. Clinical Implementation of these three methods is a progressive process while the APIC was the major progress among the three methods. Thus, we chose to compared the total number of planning errors before (including data from 2013 to 2014) and after (including data from 2015 to 2018) APIC implementation. We assigned the severity of these errors into five categories: serious (S), near miss with safety net (NM), clinical interruption (CLI), minor impediment (MI), and bookkeeping (BK). The Mann–Whitney *U* test was used for statistical analysis.

**Results:**

A total of 253 planning error forms, containing 272 errors, were submitted during the study period, representing an error rate of 3.8%, 3.1%, 2.1%, 0.8%, 1.9% and 1.3% of total number of plans in these years respectively. A marked reduction of planning error rate in the S and NM categories was statistically significant (*P* < 0.01): from 0.6% before APIC to 0.1% after APIC. The error rate for all categories was also significantly reduced (*P* < 0.01), from 3.4% before APIC and 1.5% per plan after APIC.

**Conclusion:**

With three combined methods, we reduced both the number and the severity of errors significantly in the process of treatment planning.

## 1 INTRODUCTION

The radiation oncology incident learning system (RO‐ILS)^1^, ^2^ revealed about 30% reported events occurring in the processes of treatment planning and pretreatment review/verification. Many layers of quality control (QC) are routinely embedded in external beam radiotherapy, including but not limited to physics chart review, physician plan review, therapist chart review, pretreatment measurement for intensity modulated radiation therapy (IMRT), and daily imaging guided radiotherapy. Ford et al. reported that the effectiveness of each of these QCs is <50% with an exception of the physics chart review being 67%.^3^ To achieve an effectiveness of 97%, Ford et al. recommended seven layers of QCs. As illustrated in Fig. 2 of this reference article,^4^ simplification, standardization, automation, and forced functions are top effective solutions in hazard mitigation, such as detecting errors in treatment planning.

In order to detect errors made in the planning process, research has focused on designing manual and automated checks throughout this process.^5^, ^6^, ^7^, ^8^, ^9^, ^10^, ^11^, ^12^, ^13^, ^14^, ^15^, ^16^ Automated checks focus mainly on the technical integrity of a plan and on dosimetric metrics that are established for each treatment site. Overall treatment plan quality, especially three‐dimensional dose distributions, is still dependent upon human review. A radiation therapy treatment plan should be both clinically and technically sound. An example of the clinical integrity of a plan is whether the prescription dose and dose fractionation follow a standard of care or a clinical protocol. An example of technical integrity of a plan is whether a correct computed tomography (CT) image set is used for treatment planning. The plan technical integrity can be checked using both the manual method (i.e., checklist method) and the automated computer program. Some automatic plan check methods reside outside of the treatment planning system and thus a plan check is often conducted after the treatment plan is completed.^8^, ^10^, ^11^ Therefore, if a planning error is detected, the workflow could be interrupted and rework is required, resulting in treatment delay.

In addition to the plan technical integrity, communication errors between planners and radiation therapists may also lead to mistreatment. An example of this error is whether the use of a bolus is indicated in the patient chart. These types of errors, referred to as chart errors, may not be caught during the plan integrity check using automation, thus a manual check using a checklist is needed. Our 6 yr experience indicated that we need all three approaches, combining the APIC with semi‐automatic creation of standardized plan documents and checklist method to reduce planning and chart errors, echoing simplification, standardization, automation, and enforced functions.^4^ Over the years, we implemented the checklist method and also developed a computer program named as automatic plan integrity check (APIC) within the treatment planning system. The APIC program can be called frequently when a planner is setting up the plan (e.g., placement of an isocenter) and entering the planning parameters (e.g., selection of beam angles and collimator angles). We also developed a program to semiautomatically create a standard plan report and enforce the use of standard beam names, prescription names, and the APIC program prior to the plan approval. The standard plan report facilitates other clinical staff (e.g., radiation oncologists and radiation therapists), who may not be familiar with the treatment planning system, to conduct their parts of plan approval or pretreatment plan/chart review. The purpose of this study is to describe our experience of developing these three methods. As clinical Implementation of these three methods is a progressive process and the APIC was the major progress among the three methods, we thus compare the frequency of planning errors made before and after our APIC was introduced.

## 2 MATERIALS/METHODS

### 2.A. Standard plan document format

**Fig. 1:**
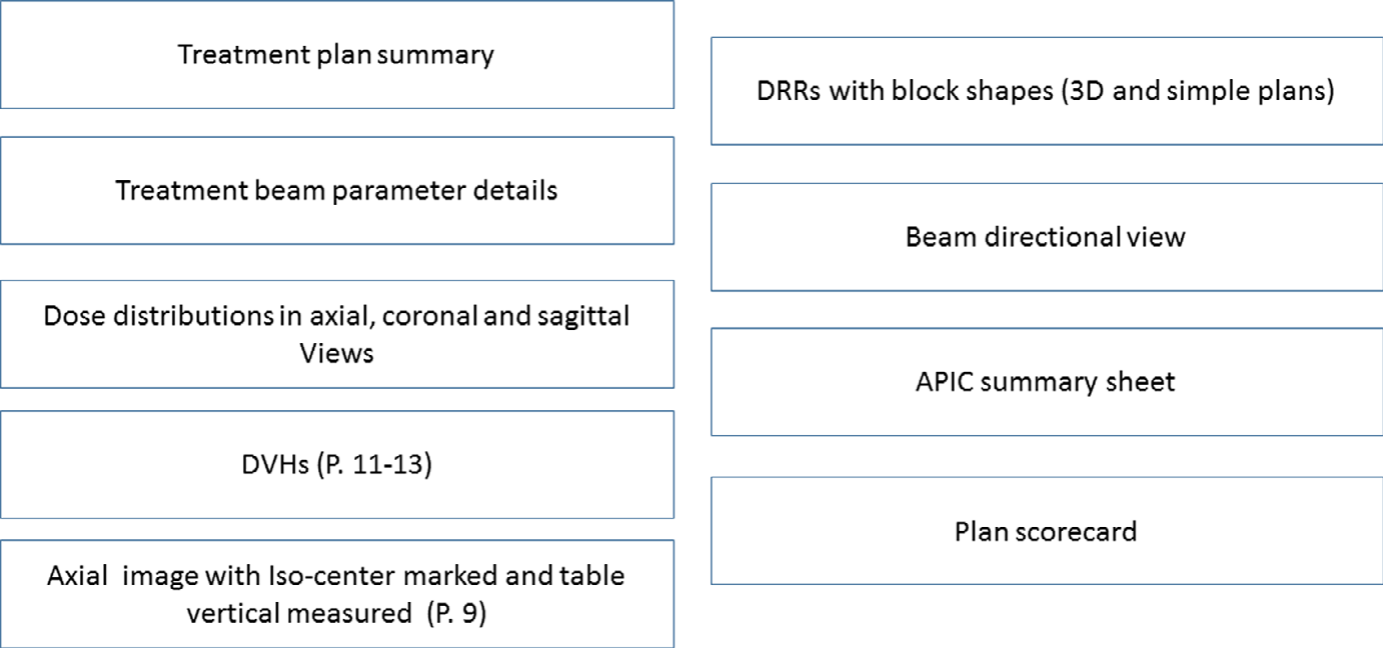
The general content orders of our treatment plan documents.

We established a standard plan document format to facilitate plan check by different team members during initial chart checks, chart rounds, or weekly chart checks. The general contents of a plan report are depicted in Fig. 1. The plan summary information and detailed information for each beam are the default format from the commercial planning system (Version 9.10, 16.2, Pinnacle, Philips Medical Solutions, Cleveland). Isodose distributions, displayed on eight axial, eight coronal, and eight sagittal images, and dose volume histograms (DVHs) for all contoured structures are screen captured. To visualize the location of the isocenter, we include an axial image with the isocenter clearly marked along with a measurement of the treatment table vertical on this image. This general format varies slightly among various plans: simple plans and/or those for urgent treatment, three‐dimensional (3D) conformal plans, intensity modulated radiotherapy (IMRT) plans, or stereotactic body radiotherapy (SBRT) plans. The plan document is simplified for less complicated and urgent plans that typically use AP, PA, both AP/PA beams, opposed lateral beams (e.g. whole brain radiation), or an en face electron beam. The simplified plans contain only dose distributions on one axial, coronal, and sagittal image at the isocenter, instead of eight axial, coronal and sagittal images covering the planning tumor/target volumes (PTV). The block shape projected on the digitally reconstructed radiograph (DRR) of each beam is included only for simple plans and 3D plans. For SBRT plans, the plan conformity index (CI, defined as a ratio of the volume encompassed by the prescription isodose line and the PTV) and R50 (defined as a ratio of the volume encompassed by 50% of the prescription isodose line and the PTV) are automatically calculated and reported in the plan document. To ensure these standard formats are used by all planners consistently, we developed three separate script menus to generate 3D, IMRT, and SBRT plan reports.

**Fig. 2:**
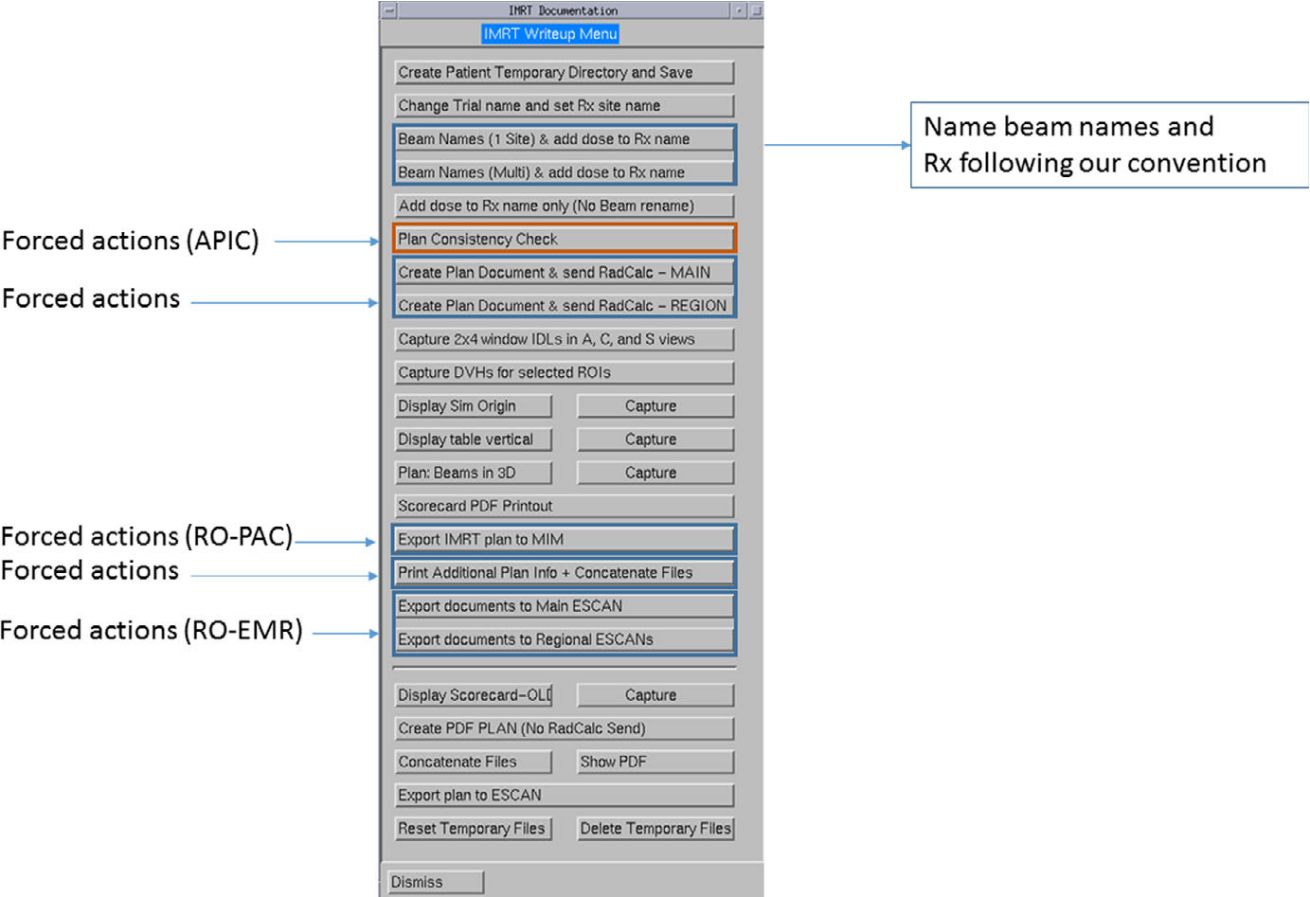
An in‐house created panel for an intensity modulated radiation therapy plan document creation.

Figure 2 shows the buttons (or steps) to create an IMRT plan report using one of the three script menus created to facilitate compliance of standard documentation. As seen In Fig. 2, not all buttons are used for creation of the plan report; some buttons are used for forced actions (as indicated in Fig. 2). One button is to run the APIC program, described in section (c), forcing each planner to run the APIC program prior to preparing the plan report. Another enforcing action button is to transfer the plan parameters to the secondary dose calculation program. There are additional buttons that force planners to send plan parameters to the RO‐EMR system, and DICOM files of planning CT, structures, and doses to the RO‐PAC system (we use MIM as our RO‐PAC system).

**Fig. 3:**
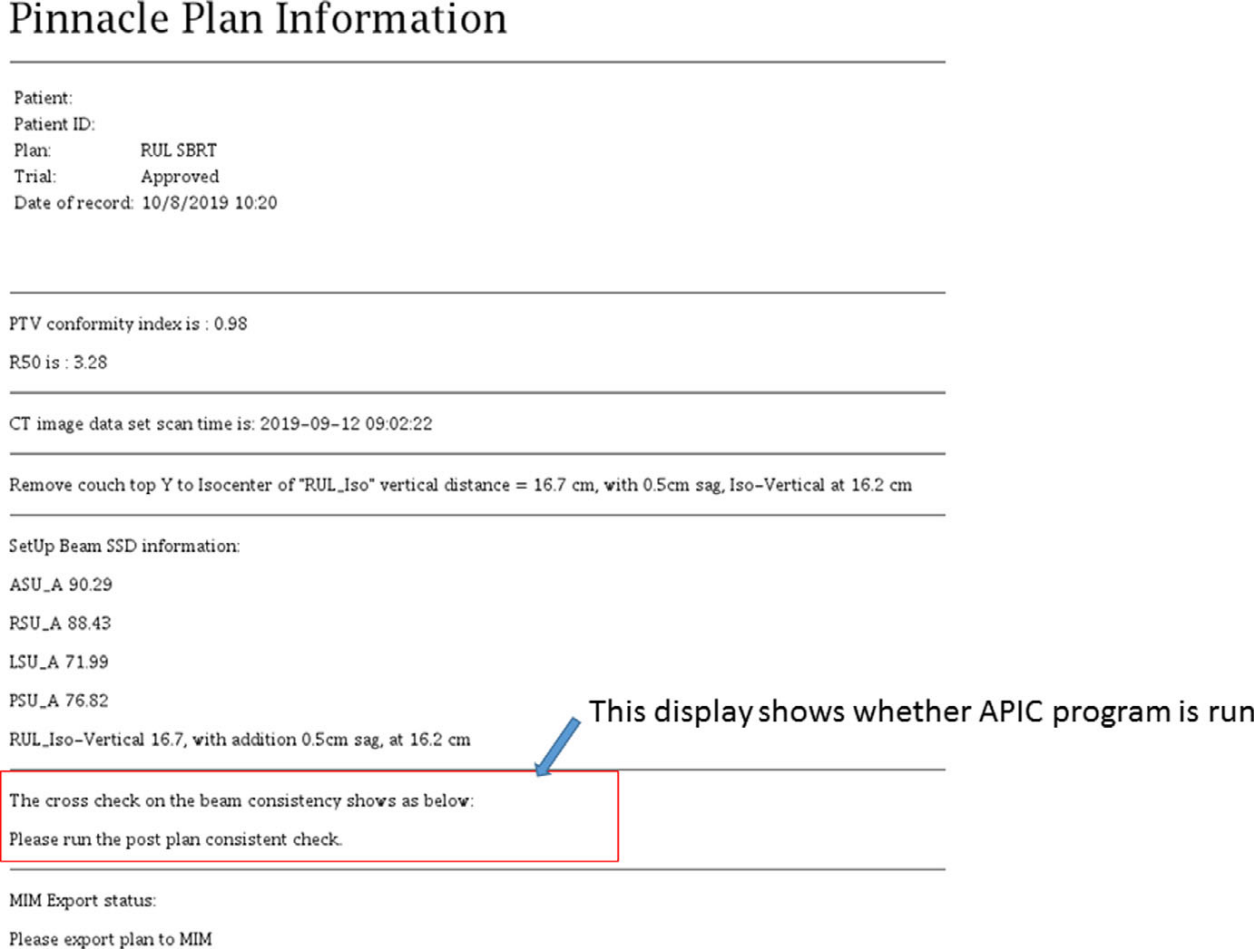
A supplement information sheet created from running Automatic Plan Integrity Check (APIC), displaying the CI and R50 (for stereotactic body radiotherapy plan only), the time‐stamp of planning computed tomography, the calculated table vertical, SSDs for all beams, and the summary result of APIC.

We use automation to avoid bookkeeping errors. For example, we established a beam naming convention which names static beams by gantry angle and names VMAT beams with starting to finishing arc angles. For noncoplanar beams, table angles are also included in the beam names. This convention is enforced following a subscript (shown in Fig. 2) that automatically creates beam names according to our local convention. The setup beams are also automatically created from the four orthogonal directions (0°, 90°, 180°, 270°). To make the treatment prescription clearly visible, we establish a standard name convention for the plan prescription which includes the total dose and treatment site. Using another subscript, the planner is prompted to enter the planned total dose and treatment site into the script which then sets the prescription name to the standard format. To facilitate checking the coordinates and location of an isocenter of the plan against the simulation document, we use another subscript to automatically locate the axial image that contains the isocenter, which is documented in the plan report. On the same axial image, the couch vertical is measured from the isocenter to the treatment couch top, which is manually defined by a red‐line that indicates the treatment table top at the early stage of the planning. In our practice, we determine the couch vertical from the plan, which is a reliable treatment parameter used for patient setup verification. The measured table vertical is compared against the calculated table vertical using a subscript embedded in the plan report script. Another subscript embedded in the plan report script is to display the CT acquisition time stamp (shown in Fig. 3). To avoid transcription errors of SSDs from the setup beams that are typically displayed with SSDs of other treatment beams, which may be misread, we use a subscript to grab these SSDs from the plan summary sheet and display them separately in the end of the plan document (shown in Fig. 3).

#### 2.A.1. Checklist methods

A new plan/chart consists of a treatment plan report, verification images, treatment parameters, and patient setup instructions. Checklist methods are effective for detecting communication errors or missing information. In our department, if a communication error or missing information stems from planners, this type of error is considered planning related errors. We use a planner checklist and a physics checklist to ensure that verification images, treatment parameters, and patient setup instructions are completed and transmitted to the RO‐EMR system. First, we use a planner checklist shown in Table 1. Upon physician approval of the plan, the planners must assemble the new charts following the checklist in Table 1. In our practice, planners also enter SSDs for either the treatment fields and/or setup fields in a document labeled as the SSD document, which is also used by therapists to record weekly SSD checks. The last section on the planner checklist is specific to our workflow. Once the new plan/chart document is completed, a quality checklist (QCL) item is created to assign a physicists to check the new plan/chart. Table 2 is a checklist used by physicists for checking the new plan and chart. These checklist methods have been in use since 2011. After a script developed for standardized documentation, the checklist for planners has been shortened to the current form in Table 1.

**Table 1:** Planner Checklist used to prepare a new chart.

Sections	Tasks	Description
RO‐EMR	Enter key parameters in Rx, CBCT, ABC/SDX, VisionRT	Frequency of CBCT acquisition and motion management methods included in Rx
	Annotate motion management (ABC/SDX) Annotated on Plan Doc	Annotation to assist chart check
	Create fields for CBCT and setup fields	
	Insert Table Vertical in each field	Table vertical measured during planning
	Assign table tolerance	
	Associate DRRs and set to review required	Specific to our RO‐EMR
	Complete/approve site set‐up	
	Amend patient setup instruction (add motion management instruction)	Patient setup instruction is initially created at simulation and then amended by a planner
	Check the origin marked on CT sim agreed with the plan	
	SSD ‐ triangulation not at the origin	Provide AP SSD at the origin under this condition
	Document Bolus in Rx, Fields, and setup notes	Bolus documented in three places: RX, field name, and setup notes
Documents	Complete SSD Doc. with table vertical range	SSD and table vertical are copied into patient setup instruction
	Complete Tx Planning Doc.	
	Create MOSFET/electron output QCL and Doc.	
Image guidance	Completed/approved CBCT Req	
	Appropriate contours in site setup	
	Send CT scan to MOSAIQ	
	Verify iso of CT and contour in site setup	
	Export external to VisionRT ( if applicable)	
Billing and plan approval	Bill for treatment plan	
	Create IMRT QA QCL	
	Page MD to sign images, plan, and RX	
	Place a chart check QCL on physics	

**Table 2:** Physics checklist used by physicists to check new charts.

Tasks	Description
Rx	Check that information is complete (dose, fraction, image frequency, and any special instruction)
Laterality/Tx site correct	Specify the anatomic site and matched with simulation
Isocenter associated W/all beams	
Isocenter/sim agreement	Check agreement of the isocenter used in the plan and set at the simulation
Iso shift & point dose documented	
Rx energy, dose, IDL/depth, Fx = plan	Check agreement of prescription in MOSAIQ and in the plan
Tissue/air threshold OK	Specific to our practice
Rx & plan signed by MD	
DRRs signed by MD	
Verify SSDs	
Check setup fields	
Plan with intended Tx unit	
Correct CT used for planning	Check CT date and simulation date
CBCT: Ref CT has correct Iso	
Plan signed by planner & physics	
Field doses sum = total Rx dose	
Field names correct	
Table vertical is same for fields in Rx	
Tolerance table correct	
Field parameters OK and fields approved	
Tx calendar in & Fx correct	
RadCalc is within 5%, 3 MU, 3 cGy	
RadCalc is signed	
IMRT QA QCL created	
Dose calc charges captured	Specific to our workflow
Create a QCL for Tx machine	Specific to our workflow

#### 2.A.2. Automatic plan integrity check (APIC)

The APIC has been developed and clinically implemented since 2015 via scripting in our treatment planning system to automatically check for the most common treatment planning errors encountered in our department. Common errors include: mismatched isocenters among beams, ambiguous gantry angle (i.e. 180°) for VMAT beams, and inconsistency between beam name and beam angles. As these errors disrupt workflow and impact safety, we created a set of rules and checks (listed in Table 3) to review the planning parameters throughout the planning process in order to reduce errors.

**Table 3:** Items checked by automatic plan integrity check (APIC).

Checked items	Warning message	Notes
For VMAT plans, no beam angles starts at 180°	*VMAT beam start/stop at 180 degree is detected*	
For VMAT plans no collimator angle is set to 0°	*VMAT beam collimator angle = 0 is detected*	*Local institution policy*
All Beams are associated with the same iso	*Beam ISO inconsistency is detected*	*For plan with multiple‐isos this warning can be ignored*
All beams (including setup beams) are associated with the same machine name	*LINAC machine name inconsistency is detected*	
For Edge machine plans, the Y jaw positions should be < 10.5 cm	*For Edge plans, the Y jaw position> 10.5 cm is detected*	*Field size limits for Edge and Novalis TX machines*
For IMRT/VMAT plan, the X yaw position should be < 14. 5 cm	*For IMRT/VMAT plan, the X jaw position> 14.5 cm is detected, which may be a problem if you use non‐variable jaw machines*	*Specific to Varian Machines*
For SBRT plans, if the couch is inserted:		*Local institution policy*
The couch removable coordinate need to be> 25 cm	*For SBRT plan with table override, please make the “remove couch from scan line” below the inserted couch table*	*These tasks are now completed by a script*
The inserted Table density override is set to 0.35 g/cm^3^	*For SBRT plans, the table override density needs to be 0.35 g/cm^3^*	
The outside patient density threshold is defined	*For SBRT plans with table override, the outside patient density should be < 0.35 g/cm^3^ or less, noncompliance is detected*	
All beams are associated with the same reference point	*Reference point inconsistency is detected*	
For non‐coplanar plans, the beam name with the non‐zero couch angle should contain a letter of “T”	*For non‐coplanar plan, a beam with the non‐zero couch angle should have its name contain a letter of “T”*	*This task is now completed by a script*
All treatment beams (excluding set up beams) have the same dose rate	*Treatment beam dose rate inconsistency is detected*	
Beam names should match the gantry or table angles. (If couch ≠ 0, the couch angle should be appeared in the beam name)	*Inconsistency between the beam name and the gantry or table angles is detected, use standard name convention*	*This task is now completed by a subscript that create plan document*
The isocenter shifts should be documented in the MOSAIQ	*Iso center shifts are detected. Please document the shifts in MOSAIQ*	
If the plan name is SBRT, please check (a) dose grid used is 0.3 cm and dose calculation method is “CCC”	*For SBRT plan, the dose grid resolution needs to be 0.3 cm and use CCC dose algorithm*.	*Local institution policy*
The treatment beam ID should be matched with the first two letters of the beam name	*Inconsistency between the beam name and the field ID is detected*	*This task is now completed by a script that creates the plan document*
Non coplanar beams, the table angles must be safe to avoid gantry collision	*A possible table and gantry collision is detected*	
Check Output Factor setting for photon and electron plans	*Inconsistency in output factor setting detected*	
Setup beam names and gantry angles are incorrect	*Setup beam name and Gantry Angles are incorrect if patient is supine*	*This task is now completed by a subscript that create plan document*
Check maximum Lateral offset at Table vertical to prevent collision	*Check table clearance. Max lateral offset may be too large with table vertical*	
Check for movement of closed leaves to prevent undeliverable beams	*One or more beams is undeliverable due to “Static” leaf gap applied to moving closed leaf line during delivery: [List of bad beams]*	
Check for MLC leaves moving beyond jaw limits	*MLC travel range maxed out: [List of bad banks and control points]*	
Check that the outside‐patient air threshold is in the proper units	*Outside‐patient air threshold not in g/cm^3^*	
Check if 180° beam angle is used in the plan	*If tumor is located on the right (patient supine), change 180° to 180.1°*	*180.1° force*s *the gantry to rotate counterclockwise*
Check any contours (except external contour) are outside of the dose grid	*List contours are outside dose grid*	
Check density overrides	*List any density overrides (inside or outside contours)*	

The items checked by the APIC program have increased from 15 items initially to the current list of 27 items (Table 3). New items are added as new planning errors are discovered. For example, we set the air threshold of 0.6 g/cm^3^ to define the external skin of a patient body. We discovered a new error whereby a planner may accidentally change the units of this default value from g/cm^3^ to CT#, which could affect accuracy of dose calculation. Upon discovering this planning error, we added an item in the APIC to check the air threshold value and units. A related issue was noted for SBRT plans, where due to a change of our treatment couch requiring us to account for treatment couch attenuation, the air threshold is set to 0.34 g/cm^3^. A frequent error resulted when a planner forgot to either insert a couch model to take into account the table attenuation or to change the threshold to 0.34 g/cm^3^, which included the couch model in the dose calculation. Discovering this error, we developed a separate script to both enable couch model insertion and change of air threshold simultaneously by one click of the script button, and added a new item to the APIC program checks for SBRT plans.

The APIC is written in and around our treatment planning system using a combination of Perl v5.8 ‐ v5.12, Python v2.6,^17^ and the internal Pinnacle^3^ treatment planning system scripting language (Koninklijke Philips N.V., Amsterdam, Netherlands). The APIC may be called at any time during the planning process to identify any issues that must be rectified before a plan is completed. Upon program call, the APIC runs though and automatically checks conditions based on the plan files and parameters without requiring further user input. The final plan document includes a record indicating the completion of the APIC and any resulting output warning messages. For a plan containing two different isocenters, a warning message of “beams are associated with different isocenters” remaining after the APIC is acceptable. For a plan with the isocenter shifted from the original isocenter set at the simulation, a warning message of “an isocenter shift is detected” is acceptable. This message will prompt the planner to document the isocenter shift in the patient setup instruction and prompt the physicist to check whether such instructions are appropriately carried out. Fig. 3 is the supplementary information sheet created from running APIC, displaying the CI and R50, the time‐stamp of planning CT, the calculated table vertical, SSDs for all beams, and the summary result of APIC. If a planner did not run the APIC during the plan document write up following the subscript shown in Fig. 2, the summary sheet of APIC would be missing or the summary sheet would display “cross check on beam consistency did not run”. During physic plan check, a physicist uses this summary sheet to ensure the APIC program was called and all warning messages have been addressed.

#### 2.A.3. Describing the workflow enhancement reporting system

In our department, we have instituted a voluntary reporting system to record any near misses, treatment plan parameter errors missed during physics check, or any workflow deviations from our practice standard. This system is called workflow enhancement reporting system (WE Form). For this study, we collected and categorized all the WE Forms labeled with “Chart Parameter Incorrect” as the source for the reported issue between January 2013 and December 2018. We compared the total number of planning errors before (2013–2014) and after (2015–2018) implementing the APIC. The severity of these errors were manually assigned by two of the authors independently (XP and LD) into five categories: serious, near‐miss with safety‐net, clinical interruption, minor impediment, and bookkeeping. The detailed definitions of these five categories are listed in Table 4. The Mann–Whitney U test was performed using R^18^ comparing reported chart errors before APIC and after APIC.

**Table 4:** Definitions of five categories of wrong chart parameters.

Categories (weight)	Description	Examples
Serious	Potential patient harm if not caught	Mismatched isocenters among all beams*
		Planned for 8Gy but Rx in R&V to 7 Gy#
		Wrong planning CT used for planning*
		A wrong plan sent to R&V#
		Energy was manually changed in R&V to a wrong energy^
Near‐miss with safety‐net	Potential patient harm but can be caught by a safety net in the process	Incorrect isocenter shifts #^
		Wrong field DRRs*
		Missing iso‐shift instruction#
		Missing Bolus information in patient setup instruction #
		Isocenter did not marked on patient correctly and did not detected by either physicist or dosimetrist#
		Use of ABC is not in patient setup#
		Setup fields were wrong*
		Wrong electronic cut out#
		Extra open MLC leaves #
Clinical Interruption	Stops the clinical process until cleared	Plan or RX not signed by MD#
		Field changed and approval revoked but not communicated with therapists#
		Mis‐spelled patient name and prevented reference CT input^
		Missing setup fields*
		Wrong dose rate#
		Wrong machine *#
		Use 180° in a VMAT plan*
		Wrong table vertical or not all fields have the same table vertical#
		Incorrect patient info sent to visionRT#
Minor Impediment	Does not stop the clinical process	Missing SSDs or wrong SSDs*
		Rad Cal is not signed#
		Wrong or missing treatment calendar#
		DRRs is not approved#
		180° (instead of 180.1°) beam angle was used for the right side tumor and may cause collision*
		Missing a block code in the field#
		Wrong or missing a tolerance table in the fields#
		Plan is not approved by a physicist#
Book‐Keeping	Mislabeling of items otherwise correct in the plan	Mismatched beam names and beam angles*
		Incorrect field order #
		CBCT is not in Rx#
		Treatment fields not listed in patient setup instruction#
		Wrong technique in Rx#
		Radcalc doc. had incorrect field names#

The examples labeled with symbol * can be detected by the APIC program; the example labeled with symbol # can be discovered by using checklist methods. Two examples labeled with symbol ^ were corrected by changing our workflow.

## 3 RESULTS

**Fig. 4:**
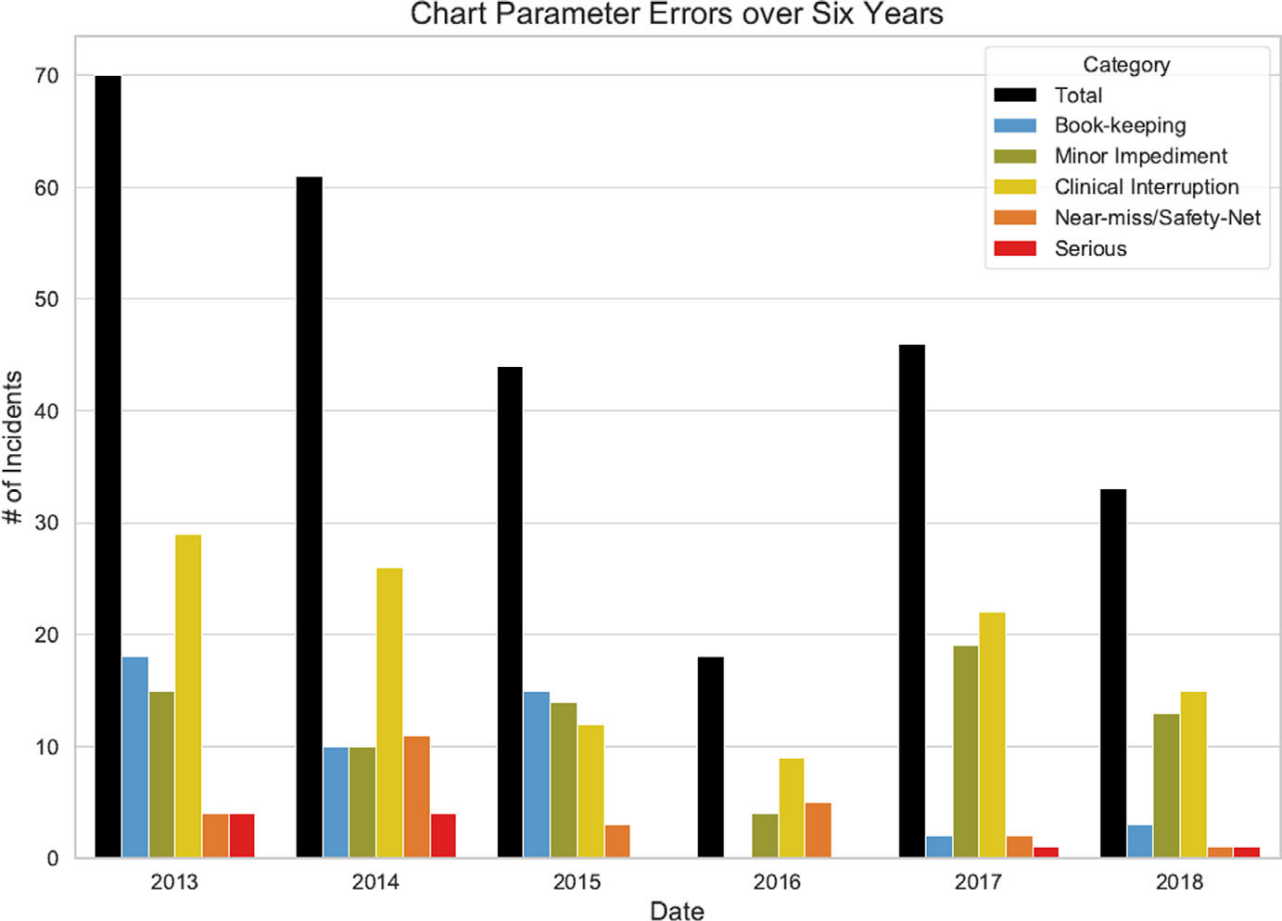
The planning errors from 2013–2018, displayed in five categories.

A total of 253 planning parameter error forms were submitted between the years of 2013 and 2018, containing 272 separate issues. During this period, the total number of new plans increased from 1843 in 2012 to 2575 in 2018, with an average increase of 7% per year. Figure 4 shows the error distributions among the five categories and the total number of reported plan errors over the six year period, representing an error rate of 3.8%, 3.1%, 2.1%, 0.8%, 1.9%, and 1.3% of total number of plans in these years, respectively. The plan error rate for the “serious” and “near‐miss” categories was significantly (*P* < 0.01) reduced from 0.6% before APIC to 0.1% after APIC. The plan error rate for all categories was also significantly reduced (*P* < 0.01) from 3.4% before APIC and 1.5% after APIC.

In the last column of Table 4, we list 37 error examples including all categories. The examples labeled with symbol * can be detected by the APIC program; the examples labeled with symbol # can be discovered by using the checklist methods. Two examples labeled with symbol ^ were corrected by changing our workflow. One of the two examples is “incorrect isocenter shift.” In our current workflow, isocenter shifts must be copied and pasted to the patient chart in the RO‐EMR system. The other error is “misspelled patient name.” In our current workflow, patient names are directly input from the RO‐EMR system to the simulation system and then to the treatment planning system.

As shown in Fig. 4, the total reported planning errors dropped by 24.1% from 2014 to 2015 and further dropped by 59.1% from 2015 to 2016. In 2017, five of six treatment machines were replaced with new models, requiring changes in the planning processes. This correlated with an increase in reported total planning errors from 18 in 2016 to 46 in 2017 with most being classified as “clinical interruption” or “minor impediment.” New APIC conditions were developed and implemented through 2017 to reflect the new machines and new workflow. Total reported planning errors once again reduced by 28.3% to 33 in 2018.

## 4 DISCUSSION

Treatment plan review and pretreatment chart check are time‐consuming processes for medical physicists. Even assuming perfect performance, the effectiveness of a physics chart check is only 60%, clear evidence that a single layer check is insufficient.^3^ Our six year experience indicated that we need all three approaches, combining the APIC with the semiautomatic creation of a standardized plan document and checklist method to reduce planning and chart errors. To compare the present study to other published studies, we listed certain features from these studies and ours in Table 5. In these studies, all used their in‐house developed programs to check certain items via automation and other items via manual process, depending on the specific treatment planning system and RO‐EMR system used in these institutions.

**Table 5:** Comparison of the present study with other published automatic plan check approaches.

	Treatment planning system (TPS)	Creation of Standard Plan report	Integration with TPS	Use by planners	Forced Function	Check Plan/chart
Breen et al^7^	Pinnacle	No	Yes	Yes	No	Plan only
Covington et al^8^	Eclipse	No	Yes	Yes	No	Yes
Dewhurst et al^9^	Pinnacle	No	Yes	Yes	No	Plan only
Halabi et al^10^	All systems	No	No	No	No	Yes
Li et al^11^	Pinnacle/Eclipse	No	No	No	No	Yes
Liu et al^12^	Eclipse	No	Yes	Yes	No	Yes
Olsen et al^14^	Eclipse	Yes	No	Yes	Yes	Yes
Berry et al^`^ ^15^	Eclipse	No	Yes	Yes	No	Yes
Furhang et al^16^	All systems	No	No	No	No	Yes
This study	Pinnacle	Yes	Yes	Yes	Yes	Yes

Many have described their efforts to improve treatment planning workflow, which varies significantly among institutions. Olsen et al. from Washington University described their automatic workflow in treatment planning to minimize manual entries while improving consistency and efficiency.^14^ At our institution, physicians use the MIM system (MIM Software Inc. Cleveland, OH, USA) to contour. We established a workflow to automatically call a treatment site specific template once a simulation CT tagged with the designated site is received in the MIM system. In the treatment site specific template, the standard names for the tumor volumes and organs at risk are loaded for contouring. We utilize the scorecard function in the Pinnacle treatment planning system to evaluate plan quality in real time. In addition to the use of a checklist for planners at the final stage of planning and a checklist for physicists during the initial plan check, we use automation to create a standard plan document and use the APIC system to check certain planning parameters at multiple stages of planning, catching errors early in the process.

The importance of pretreatment planning and chart checking has prompted many institutions to develop in‐house programs to audit and check planning process, which is labor intensive with time constraints.^5^, ^6^, ^8^, ^10^, ^12^, ^13^, ^19^ Berry el al. from Memorial Sloan Kettering Cancer Center (MSKCC) reported their five year experience of deploying a customized electronic checklist for quality assurance of treatment planning.^6^ Covington et al. described a plan check tool developed at University of Michigan to automatically evaluate plans,^8^ noting a 60% reduction in patient delay after instituting their plan check tool. Interestingly, they found that the number of errors found during the physics initial plan check did not decrease, rather the automatic plan check heightened error visibility.

Using a similar approach to ours, Breen et al. in 2010 reported that using an automatic checklist during planning halved the plan rejection rates from nearly 6% to 3%.^7^ They also pointed out that the compliance with using an automatic checklist was low, necessitating a forced function. In our process, we require each planner to include the summary page of APIC attached to the plan report. If the planner did not run the APIC, either the APIC summary sheet would be missing or a sentence of “APIC did not run” would display on the summary sheet at the end of the treatment plan document, which would prompt a plan check physicist to return the chart back to the planner, enforcing the action. Often, a planner will run the APIC multiple times, prior to optimization, prior to the plan review with physicians, and prior to preparation of the plan document. Most planners at our practice reported that they use the APIC program multiple times to prevent errors upstream and eliminate wasting time to rework.

Using scripting language in the Pinnacle system, Dewhurst et al. reported their semi‐automated plan check system with an action rate of 30% by the planners after running their Auto‐Lock system, indicating a significant reduction of potential errors.^9^ Our error reduction analysis includes plan errors and charts errors discovered by therapists during their new plan/chart check, prior to treatment. One of the frequent errors discovered in new chart checking by therapists is the mislabeling of beams and setup images. To reduce these types of errors, we introduced a standard plan documenting script, which automatically labels the beam and beam IDs according to our local conventions and automatically generates orthogonal setup beams. Transcribing a wrong SSD from the planning document to patient setup instruction is another common chart error. As these types of errors cannot be automatically detected by the APIC program, they become checklist items that are checked by physics during a new chart check.

One of the limitations of the APIC program and semiautomatic plan document creation script is that it is not integrated with the RO‐EMR system. The manually entered communications between the planning system and RO‐EMR system can only be checked by the checklist method. Using an integrated planning system and RO‐EMR system, Liu et al from Stanford University reported several items listed in our checklists can be automatically checked.^12^ We are in the progress to develop another program in our RO‐EMR system to automatically check certain items listed in our checklists. Even with the integrated planning system and RO‐EMR system, some communication errors listed in Table 4 still require manual checks such as the use of bolus and instruction of isocenter shifts. A recent publication from MSKCC, under the integrated environment of the treatment planning and RO‐EMT systems, indicated that their automatic plan check tool reported three outputs for their checked items: pass, flag, and report.^15^ The items labeled with “flag” or “report” require manual check. Using the APIC to eliminate certain planning errors, planners and physicists can spend more time to check communication errors or chart errors. We hope that the effectiveness of the APIC demonstrated in this study should inspire vendors to develop a commercial program for common users to perform plan integrity check.

## 5 CONCLUSION

With reporting incidents involving incorrect chart parameters, we performed thorough investigations and identified underlying causes. With the use of an automatic plan consistency check (APIC) program, augmented with standard plan documentation and checklist methods, we demonstrated successful reductions of the number and severity of errors in the process of treatment planning.

## CONFLICT OF INTEREST

Authors have no conflict of interest to disclose.
